# Development and validation of a novel 15‐CpG‐based signature for predicting prognosis in triple‐negative breast cancer

**DOI:** 10.1111/jcmm.15588

**Published:** 2020-07-10

**Authors:** Yang Peng, Lin Shui, Jian Xie, Shengchun Liu

**Affiliations:** ^1^ Department of Endocrine and Breast Surgery The First Affiliated Hospital of Chongqing Medical University Chongqing China; ^2^ Department of Medical Oncology Cancer Center West China Hospital Sichuan University Chengdu China; ^3^ Department of General Surgery Yongchuan Hospital of Chongqing Medical University Chongqing China

**Keywords:** methylation sites, nomogram, prognosis, TCGA, triple‐negative breast cancer

## Abstract

DNA methylation is an important biological regulatory mechanism that changes gene expression without altering the DNA sequence. Increasing studies have revealed that DNA methylation data play a vital role in the field of oncology. However, the methylation site signature in triple‐negative breast cancer (TNBC) remains unknown. In our research, we analysed 158 TNBC samples and 98 noncancerous samples from The Cancer Genome Atlas (TCGA) in three phases. In the discovery phase, 86 CpGs were identified by univariate Cox proportional hazards regression (CPHR) analyses to be significantly correlated with overall survival (*P* < 0.01). In the training phase, these candidate CpGs were further narrowed down to a 15‐CpG‐based signature by conducting least absolute shrinkage and selector operator (LASSO) Cox regression in the training set. In the validation phase, the 15‐CpG‐based signature was verified using two different internal sets and one external validation set. Furthermore, a nomogram comprising the CpG‐based signature and TNM stage was generated to predict the 1‐, 3‐ and 5‐year overall survival in the primary set, and it showed excellent performance in the three validation sets (concordance indexes: 0.924, 0.974 and 0.637). This study showed that our nomogram has a precise predictive effect on the prognosis of TNBC and can potentially be implemented for clinical treatment and diagnosis.

## 1 INTRODUCTION

Breast cancer (BC) is a main health burden for women worldwide. There is an increasing incidence of BC throughout the world, and it has become the main cause of mortality and morbidity in females.^1^, ^2^ Triple‐negative breast cancer (TNBC) is an aggressive subtype of BC that is defined as the absence of oestrogen receptor, progesterone receptor and HER2 expression.^3^, ^4^ These patients lack corresponding therapeutic targets and have high recurrence and mortality compared to patients with other subtypes of BC.^5^ Although TNBC is highly malignant, it is still necessary to avoid overtreatment in cancer patients.^6^ Therefore, it is particularly important to understand the pathogenesis and mechanisms of TNBC and to find corresponding therapeutic targets.

DNA methylation is an important biological regulatory mechanism that changes gene expression without altering the DNA sequence.^7^ Increasing studies have revealed that DNA methylation data play a vital role in the field of oncology.^8^, ^9^ In addition, the emergence of high‐throughput technology makes it possible to identify reliable markers. Several studies have reported that DNA methylation may play a key role in predicting prognosis in various cancers.^10^, ^11^, ^12^ For example, a DNA methylation signature was identified by Sandoval et al that improved prognostic accuracy beyond standard staging in non‐small‐cell lung cancer,^12^ and Lasseigne et al proposed novel methylation‐related biomarkers. In their study, DNA methylation profiles were used to distinguish between tumours and benign adjacent tissues of renal cell carcinoma.^11^ In BC research, Monika Lesicka et al showed that some circadian genes with abnormal methylation patterns may be novel indicators and may play an important role in BC aetiology.^13^ Bin Xiao et al identified several major methylation sites for predicting the prognosis of BC in the luminal subtype.^14^

However, to the best of our knowledge, few studies have investigated the prognostic value of methylation sites in TNBC. Therefore, this study was designed to investigate overall survival (OS)‐related CpGs in TNBC. First, we selected 86 candidate CpGs in 120 training samples; we then validated those CpGs in 38 testing samples and 37 external validation samples. Finally, a nomogram incorporating the prognostic risk model and clinicopathological features was developed. Overall, our nomogram has a precise predictive effect on the prognosis of TNBC and may potentially be implemented for clinical treatment and diagnosis.

## 2 MATERIALS AND METHODS

### 2.1 Data source

Breast cancer datasets, including BC methylation, mRNA expression profiles and clinical information, were downloaded from The Cancer Genome Atlas (TCGA) (https://gdc‐portal.nci.nih.gov/). A total of 164 TNBC samples and 98 normal breast samples with methylation data (Platform: Illumina Infinium Human Methylation 450), mRNA‐Seq data (Platform: Illumina HiSeq 2000 RNA sequencing) and clinical information were downloaded for further analysis. Baseline clinicopathological data, including age, sex, race, menopausal status, margin status and American Joint Committee on Cancer (AJCC) stage, were derived from TCGA clinical data. It is worth noting that the 50‐gene signature test (PAM50)^15^ was performed to identify TNBC samples. Moreover, the DNA methylation data of the GSE75067 (Illumina Infinium Human Methylation 450) dataset, containing 37 TNBC samples, were downloaded from the Gene Expression Omnibus (GEO) database as the validation set.

### 2.2 Study design

In our study, the inclusion criteria of samples were as follows: (a) both methylation level and survival data were available; (b) OS time was more than 1 month; and (c) histologically confirmed invasive TNBC. A total of 158 TNBC samples with complete survival information were obtained. Following the methods of random sampling at a ratio of 70:30, the 158 TNBC samples were separated into the training set (n = 120) and test set (n = 38). To avoid the reduction in statistical test efficiency and the bias caused by the direct exclusion of missing values, we used multivariate interpolation to estimate the missing values^16^ (Appendix S1).

Three phases were used to investigate the OS‐related methylation sites in TNBC patients. In the discovery phase, LIMMA package has been used to carry out normalization and the Mann–Whitney U‐test was used to compare methylation differences between the TNBC and normal samples, sites with DNA methylation levels with false discovery rate (FDR) <0.05 and |log_2_ fold change (FC)| ≥ 0 were defined as differentially methylated sites (DMSs). Then, univariate Cox proportional hazards regression (CPHR) analysis was performed to select significant DMSs correlated with OS. Finally, the 86 DMSs most related to OS with *P* < 0.01 were selected for least absolute shrinkage and selector operator (LASSO) Cox regression in the training set to narrow down the candidate CpGs using the R package glmnet.

The risk score was calculated as follows:

Risk score (CpG‐based signature)=sum of coefficients×expression level of CpGs.

15 methylation sites were found to have nonzero coefficients in the model, and the optimal cut‐off value of −11.6 was derived from the time‐dependent receiver operating characteristic (ROC) curve using the Youden index. The samples with a risk score greater than −11.6 were divided into the high‐risk group, and the remaining samples were divided into the low‐risk group. Finally, the 15‐CpG‐based clinicopathological nomogram was built according to the results of the multivariate Cox analyses.

In the validation phase, we validated our nomogram in three different cohorts. The area under the curve (AUC) based on the time‐dependent ROC analysis^17^ was calculated to assess the risk scoring system. We also performed Kaplan–Meier survival curve analysis to identify its prognostic value. In addition, stratified analysis was carried out to identify whether the CpG‐based signature was correlated with OS regardless of different clinical features. The calibration curve was plotted by the rms package of R software to estimate the consistency between the prediction outcomes of the model and the actual outcomes. Harrell's C‐index was calculated to measure the goodness of fit of the CpG‐based signature nomogram.

### 2.3 Functional enrichment analysis of the differentially expressed genes between the two groups

According to the risk scores based on the 15‐CpG signature, 158 TNBC samples were divided into high‐ and low‐risk groups. The limma and DESeq2 R packages were used for differentially expressed gene selection. Genes with were recognized as significantly differentially expressed. Gene Ontology (GO) analysis and Kyoto Encyclopedia of Genes and Genomes (KEGG) pathway enrichment analysis were performed by the clusterProfiler R/Bioconductor package.

^18^

### 2.4 Statistical analysis

All statistical analyses were conducted using R version 3.6.1. The Mann–Whitney *U*‐test and the Pearson chi‐square test were performed to compare the associations of continuous and categorical variables, respectively, between the training set and testing set. Univariate and multivariate CPHR analyses were used to identify the predominant prognostic factors of OS (*P* < 0.05). Kaplan–Meier survival curves were compared using the log‐rank test. The limma R package was used to nominalize the data, and the DESeq2 R package was used to identify differentially expressed genes. The ggplot2 R package was used to plot the volcano plot and heat map. *P* < 0.05 (two‐sided) was considered statistically significant.

## 3 RESULTS

### 3.1 Patient characteristics

Our research flow chart is shown in Figure . Six samples were excluded because the OS time was <30 days. Therefore, a total of 158 TNBC samples from 888 BC samples from TCGA were included. The primary cohort was divided into a training set and a testing set at a ratio of 70:30 with the method of random sampling. The detailed baseline clinical features of the training and testing sets are shown in Table [Link], [Link]. There were no statistically significant differences between the two independent sets, as shown in Table [Link], [Link] (all *P* > 0.05). The detailed baseline clinical features of the TNBC and normal samples are also shown in Table S2.

**FIGURE 1:**
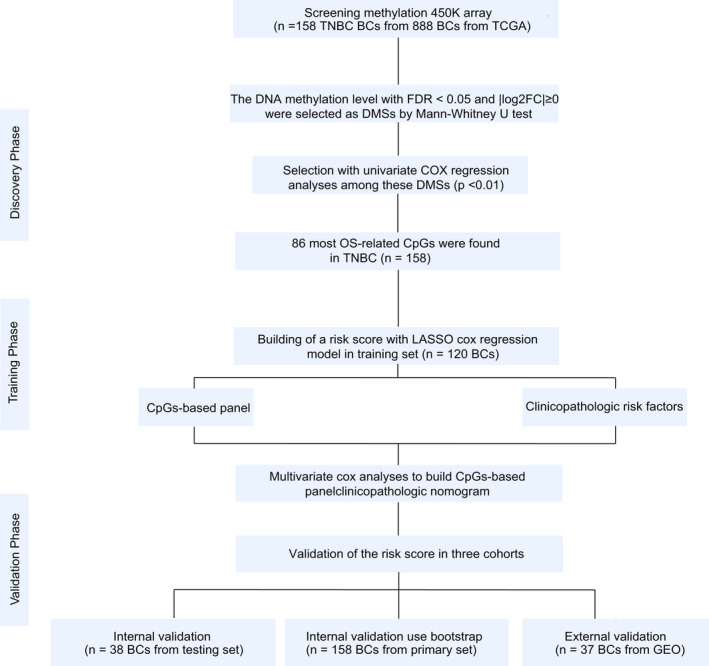
Study flow chart; TNBC, triple‐negative breast cancer; BC, breast cancer; TCGA, The Cancer Genome Atlas; DMSs, differentially methylated sites; OS, overall survival; LASSO, least absolute shrinkage and selection operator; GEO, Gene Expression Omnibus

### 3.2 Candidate OS‐related methylation sites were found in the training set

**FIGURE 2:**
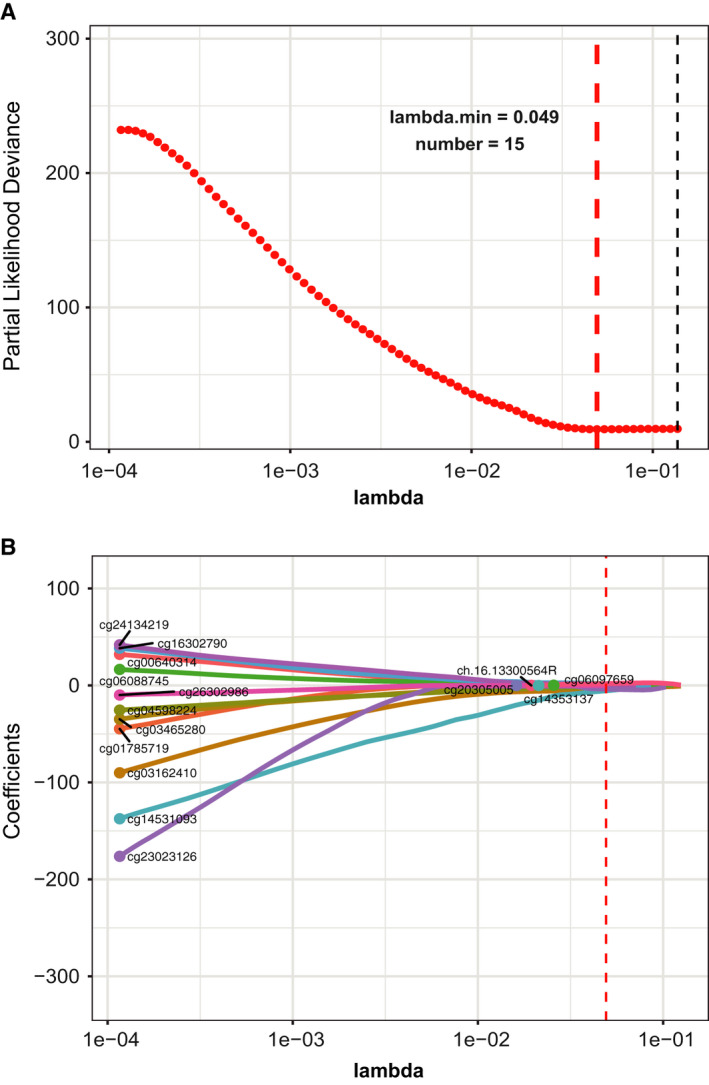
CpG selection using the least absolute shrinkage and selection operator (LASSO) Cox regression model. A, Tuning parameter (lambda) selection in the LASSO model used 10‐fold cross‐validation via minimum criteria. The partial likelihood deviation curve was plotted versus lambda. Dotted vertical lines were drawn at the optimal values by using the minimum criteria and the 1 standard error of the minimum criteria (the 1‐SE criteria). B, LASSO coefficient profiles of the 86 CpGs. A coefficient profile plot was produced against the log (lambda) sequence. A vertical line was drawn at the value selected using 10‐fold cross‐validation, where the optimal lambda resulted in 15 nonzero coefficients

The DNA methylation levels were compared between 98 adjacent normal breast tissues and 158 TNBC samples using the limma R/Bioconductor package. A total of 225153 DMSs (FDR < 0.05 and |log_2_FC| > 0) were identified (Appendix S2). Next, these DMSs were subjected to univariate CPHR analysis in the 158 TNBC samples. Then, we observed 86 methylation sites that were significantly related to OS (*P* < 0.01) (Appendix S3), and these candidate methylation sites were subsequently selected for LASSO Cox regression in the training set. Finally, 15 methylation sites were found to have nonzero coefficients in the model (Figure 2A,B).

### 3.3 Establishment of a 15‐CpG‐based prognostic model

**FIGURE 3:**
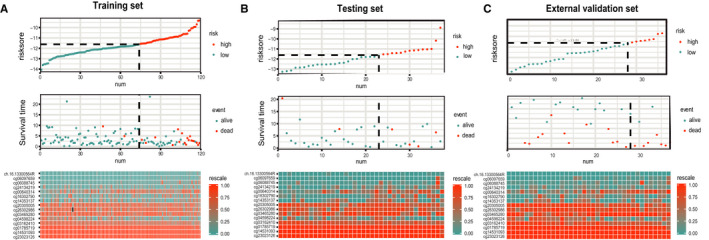
The distributions of the risk score, OS, and OS status and the heat map of the 15‐CpG prognostic signature in the training set (A), internal testing set (B) and external validation set (C). The dotted line indicates the cut‐off point of the median risk score used to stratify patients into the low‐risk group and high‐risk group. OS, overall survival

A risk score was generated to better identify the prediction efficiency of the 15‐CpG‐based signature (Figure 2B and Appendix S4). The samples with a risk score greater than −11.6 were divided into the high‐risk group, and the remaining samples were divided into the low‐risk group. The features and coefficients of these methylation sites are shown in Table S3. The distributions of the 15‐CpG risk scores, survival time, survival status and 15‐CpG expression profiles are shown in Figure 3A‐C (training set, testing set and external testing set, respectively).

**FIGURE 4:**
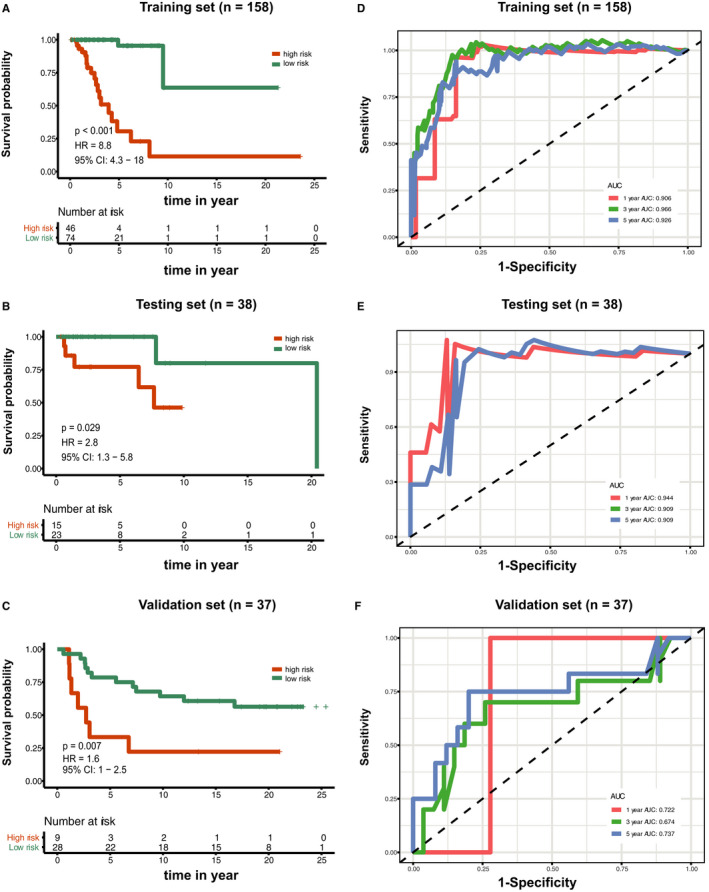
Kaplan–Meier overall survival curves of breast cancer patients based on the 15‐CpG signature in the training set (A), testing set (B) and validation set (C). Time‐dependent receiver operating characteristic curves at 1, 3 and 5 y based on the 15‐CpG signature in the training set (D), testing set (E) and validation set (F)

To identify whether the 15‐CpG‐based signature could predict OS, Kaplan–Meier survival curve analyses were conducted to show that the samples with high‐risk scores were significantly correlated with poor prognosis in the training set (*P* < 0.001) (Appendix S5), testing set (*P* = 0.029) (Appendix S6) and external validation set (*P* = 0.007) (Appendix S7) (Figure 4A‐C, respectively). Then, time‐dependent ROC curve analysis was performed. The AUC values of the 15‐CpG signature for predicting OS at 1, 3 and 5 years were 0.906 (95% CI: 0.824‐0.987), 0.966 (95% CI: 0.921‐0.999) and 0.926 (95% CI: 0.851‐0.999), respectively, in the training set (Figure 4D). We also conducted these analyses in the testing set and external validation set, and the AUC values at 5 years were 0.909 (95% CI: 0.641‐0.999) and 0.737 (95% CI: 0.542‐0.931), respectively (Figure 4E,F). Therefore, these AUC values demonstrated that the 15‐CpG‐based signature had beneficial discrimination performance for TNBC patients.

### 3.4 Assessment of the 15‐CpG signature in clinical characteristic subgroups

The results of univariate and multivariate CPHR analyses are shown in Table S4, and the 15‐CpG signature and AJCC stage were recognized as independent prognostic features. Although T stage, N stage and M stage were significantly associated with survival, they were not included in the multivariate analysis to avoid multicollinearity.^19^ In addition, to identify whether the 15‐CpG signature can predict OS regardless of different clinicopathological factors, risk stratification in TNBC patients was performed. Kaplan–Meier survival curve analyses showed that the low‐risk group was significantly correlated with better OS in T1‐T2 stage (*P* < 0.0001), T3‐T4 stage (*P* = 0.00076), N0 stage (*P* = 0.033), N1‐N3 stage (*P* < 0.0001), AJCC I‐II stage (*P* < 0.0001) and AJCC III‐IV stage (*P* < 0.0001) patients (Figure [Link], [Link]).

**FIGURE 5:**
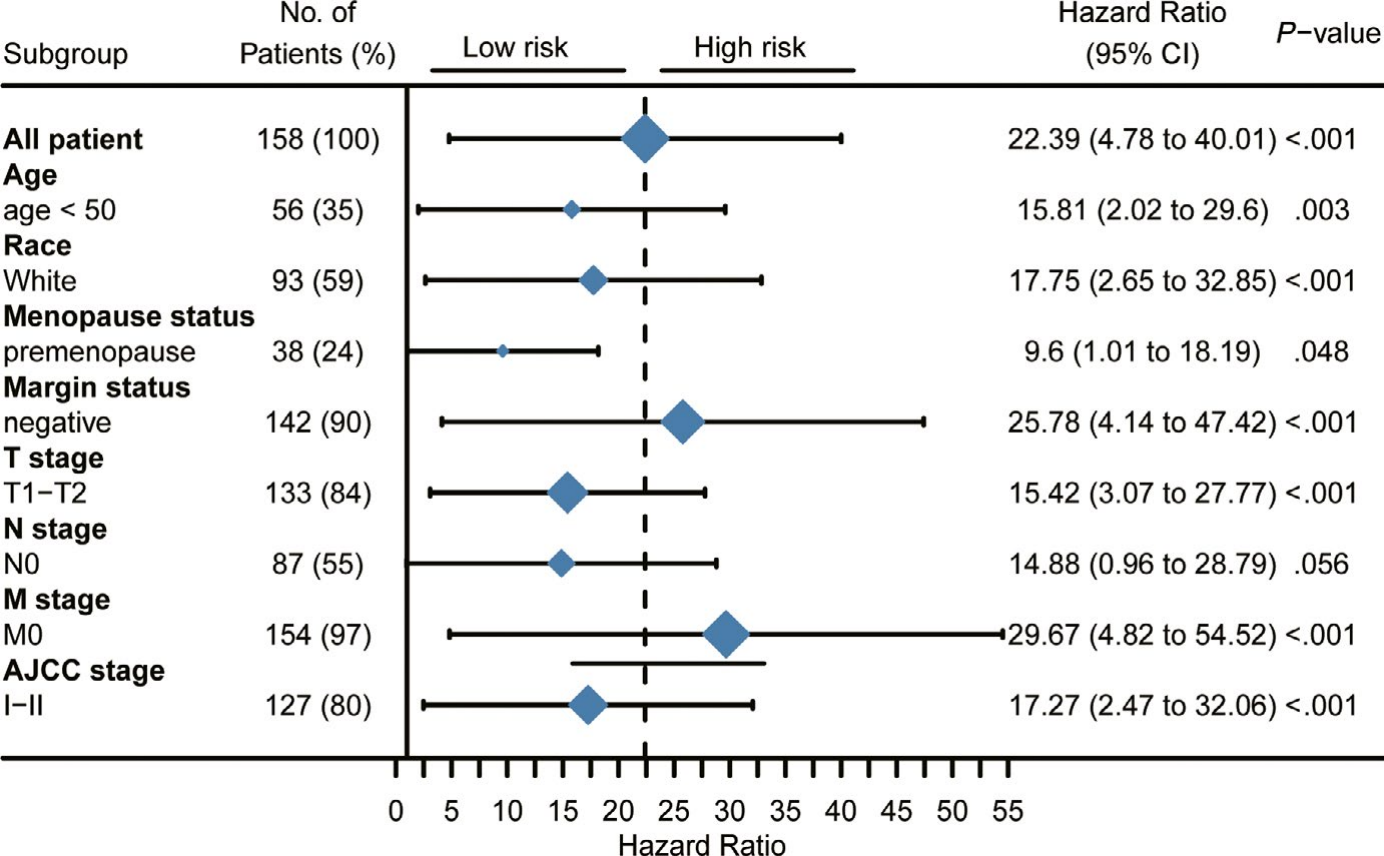
Stratified analysis of the 15‐CpG signature in breast cancer patients according to different clinicopathological subgroups. AJCC, American Joint Committee on Cancer

Upon stratification of the samples according to different clinical characteristic subgroups, OS was estimated between the low‐ and high‐risk score groups for all TNBC patients (Figure 5). Significant differences were observed in all early‐stage subgroups, including young age (HR, 15.81; 95% CI, 2.02‐29.6), T1‐T2 stage (HR, 15.42; 95% CI, 3.07‐27.77), N0 stage (HR, 14.88; 95% CI, 0.96‐28.79), M0 stage (HR, 29.67; 95% CI, 4.82‐54.52) and AJCC stage (HR, 17.27; 95% CI, 2.47‐32.06). In addition, white race (HR, 17.75; 95% CI, 2.65‐32.85), negative margin status (HR, 25.78; 95% CI, 4.14‐47.42) and premenopausal status (HR, 9.6; 95% CI, 1.01‐18.19) also showed significant differences between low and high 15‐CpG signature‐based risk scores.

### 3.5 Building a predictive nomogram

**FIGURE 6:**
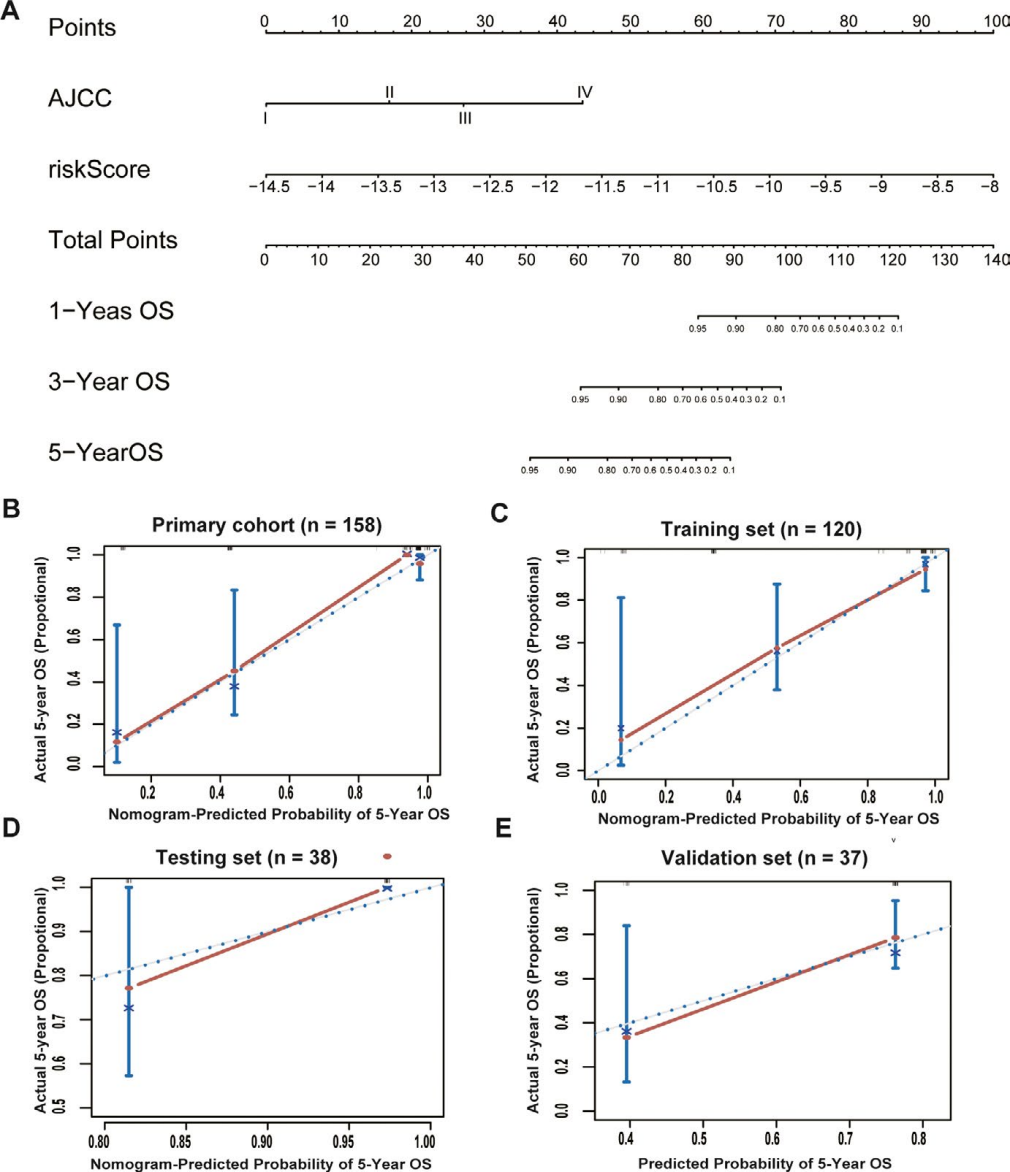
The 15‐CpG‐based prognostic model to predict 5‐y OS in TNBC patients (A). Calibration curves for the OS nomogram model in the internal primary cohort (B), internal training set (C), internal testing set (D) and external validation set (E). The blue dotted line represents the ideal nomogram, and the red line represents the observed nomogram. OS, overall survival; AJCC, American Joint Committee on Cancer

To identify the best prognostic nomogram, three models were built to compare their predictive accuracies (Table [Link], [Link]). As a result, model 1 (including risk features and AJCC stage) had a significantly better predictive performance than the other two models (C‐index: 0.918). To provide a clinically applicable method that could predict a patient's OS probabilities, these independently associated risk features were used to build a risk estimation nomogram (Figure 6A). The predictors included the risk score based on the 15‐CpG signature and AJCC stage. The calibration plots for the survival rate at 5 years showed that the nomogram performed well in the four validation sets (C‐index: 0.907 for the primary set using the bootstrap validation method, 0.924 for the training set and 0.974 for the testing set; Figure 6B‐D). In the external validation set, there was no information about AJCC stage, and the C‐index (0.637) represents the risk score based on only the 15‐CpG signature (Figure 6E).

### 3.6 Gene expression differences between the high‐ and low‐risk score groups based on the 15‐CpG signature

**FIGURE 7:**
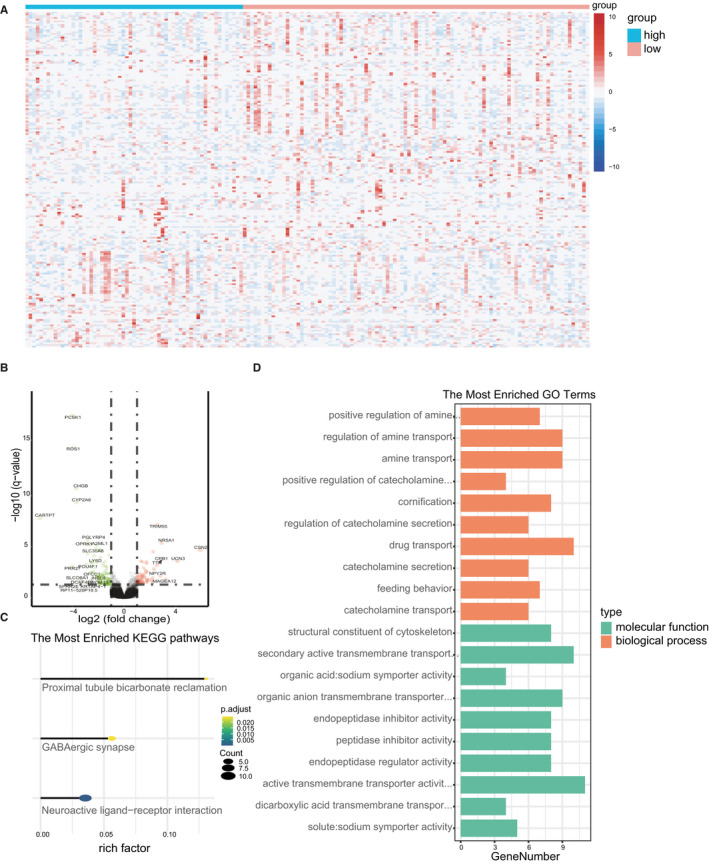
Functional annotation of genes differentially expressed between the low‐ and high‐risk groups. Hierarchical clustering analysis of the expression levels of the most related genes(A), Volcano plot of 191 mRNAs in TNBC patients. Orange colour indicates up‐regulated expression, and green colour represents down‐regulated expression (B), KEGG pathway analysis of significantly correlated genes (C),GO analysis of the most related genes (D)

According to their risk scores, the 158 TNBC samples were divided into high‐ and low‐risk groups based on the 15‐CpG signature (61 samples were in high‐risk group and 97 samples were in low‐risk group). The differentially expressed genes were selected by the limma R package. Hierarchical clustering analysis was used to show the expression levels of the genes most related to the risk scores as heatmaps (Figure 7A). A total of 191 genes that displayed significant differential expression (FDR < 0.05 and |log_2_FC| ≥ 1) in the different groups were found (Figure 7B). The results showed that 72 genes were positively correlated with the risk scores and 119 genes were negatively correlated with the risk scores (Appendix S8). To estimate the potential function of these genes, GO and KEGG pathway analyses were conducted (Figure 7C,D). The overall results of these analyses indicated that these genes may be related to material metabolism and transport.

## 4 DISCUSSION

In the era of precision medicine, a method based on molecular markers to accurately predict the survival of patients with cancer is urgently needed. A previous study showed that methylation data play an important role in the prognosis of BC patients.^14^, ^20^, ^21^ Most recently, Chuntao Tao et al found a 7‐CpG‐based signature to predict prognosis in BC patients.^20^ However, although this study used three different databases to identify prognostic biomarkers, it was not validated by any other databases. Ming Shan et al identified RASSF1a, P16 and PCDHGB7 as having significant diagnostic value for BC (AUC, 0.781; *P* < 0.001). These epigenetic markers may play a key role in diagnosing BC.^21^ Monika Lesicka et al showed that some circadian genes with abnormal methylation patterns may be novel indicators and may play an important role in BC aetiology.^13^ Bin Xiao et al identified several major methylation sites for predicting the prognosis of BC in the luminal subtype.^14^

Different researchers have identified distinct markers, indicating that there are no comprehensive biomarkers to predict the prognosis of BC. Moreover, different subtypes of BC have different methylation profiles^22^ because they could be affected by different genetic and epigenetic mechanisms.^23^ However, TNBC lacks corresponding therapeutic targets and has high recurrence and mortality compared to the other subtypes.^5^ Therefore, our research was designed to determine the prognostic value of methylation sites in TNBC.

In our research, DNA methylation data and survival data were acquired from TCGA to build a novel CpG‐based prognostic model. First, 86 CpGs were found to be significantly associated with OS by using univariate CPHR analysis. Subsequently, these OS‐related CpGs were narrowed down to 15 candidates by LASSO Cox regression analysis in the training set. Eventually, the 15‐CpG signature was validated in three internal sets and an external set. Our data identified that patients with TNBC can be separated into two groups with high or low‐risk scores based on the 15‐CpG signature, and the patients with high‐risk scores were significantly correlated with poor prognosis.

To build the best prognostic nomogram, some clinical characteristics were also analysed. In our study, only N stage, M stage and AJCC stage were significantly associated with OS. To avoid the occurrence of multicollinearity,^19^ we selected only AJCC stage for our final prognostic nomogram. The C‐index of the 15‐CpG signature and AJCC stage‐based prognostic model was 0.918, which was better than that of the 15‐CpG‐based prognostic model and the AJCC stage‐based prognostic model (C‐index: 0.819 and 0.789, respectively). Therefore, our 15‐CpG‐based nomogram may serve as a novel tool to predict prognosis in TNBC patients.

The occurrence of human diseases is caused by a complex regulatory network. Multisite methylation as a biomarker is more specific and sensitive than single‐site methylation. In this study, a total of fifteen methylation sites were identified as a prognostic signature, which corresponded to 11 protein‐coding genes (TNFRSF18, IRX3, PDX1, TCF24, SCN2A, CDK14, NACAP1, TECR, TSNARE1, ANKRD9 and MIER2). Previous studies have identified that CDK14 and ANKRD9 are correlated with cancer. CDK14 is located on chromosome 7 and participates in the occurrence and development of various malignancies, including hepatocellular carcinoma,^24^ gastric carcinoma,^25^ breast cancer^26^ and oesophageal cancer.^27^ ANKRD9 is located on chromosome 14; it acts as a receptor subunit of ubiquitin ligase substrate and is associated with tumour inhibition.^28^

Some candidate markers, such as IRX3, PDX1 and SCN2A, have not been found to be associated with tumours. However, their reported functions are similar according to our GO and KEGG pathway analyses. IRX3 is located on chromosome 16 and plays multiple roles in the pattern formation of vertebrate embryos.^29^ IRX3 deficiency partly inhibits the browning process of white adipocytes by regulating the transcriptional activity of UCP1. Rare mutations in IRX3 were correlated with obesity in humans.^30^ PDX1 plays a key role in early pancreatic development and participates in the glucose‐dependent regulation of insulin gene expression.^31^ SCN2A has been recognized as an important factor in a series of neurodevelopmental disorders.^32^

Our results also identified 191 genes that displayed significant differential expression (FDR < 0.05 and |log2FC| ≥ 1) between the two groups divided by risk score. In the GO and KEGG pathway analyses, these genes were mainly enriched in biological processes such as the regulation of catecholamine secretion and transport.

There are limitations in our study. First, it has been reported that the prevalence of TNBC is different in different races.^33^ However, our main research data were downloaded from the TCGA database, so most of the patients were white women. Whether our predictive model can be applied to non‐white female patients needs further study. Second, some methylation sites might be difficult to use as clinical diagnoses because they may not be easy to detect in serum. Third, although this study was validated using GEO data, further studies are needed to validate our research.

In summary, we built a nomogram including the 15‐CpG signature and AJCC stage to predict prognosis in TNBC patients. The performance of the nomogram was verified in different validation sets. Therefore, our nomogram may potentially be implemented to predict the prognosis of patients with TNBC.

## CONFLICT OF INTEREST

On behalf of all authors, the corresponding author states that there is no conflict of interest.

## AUTHOR CONTRIBUTION

**Yang Peng:** Conceptualization (lead); Data curation (lead); Formal analysis (lead); Software (lead); Visualization (lead). **Lin Shui:** Data curation (equal); Formal analysis (equal); Funding acquisition (equal); Software (equal); Visualization (equal). **Jian Xie:** Methodology (equal); Project administration (equal). **Shengchun Liu:** Funding acquisition (supporting); Resources (supporting); Writing‐review & editing (supporting).

## Supporting information

Fig S1

Tab S1

Tab S2

Tab S3

Tab S4

Tab S5

App S1

App S2

App S3

App S4

App S5

App S6

App S7

App S8

## Data Availability

All data sets analysed for this study are included in the manuscript and the supplementary files.
