# Supplementary figures and images for: Development and validation of a novel 15‐CpG‐based signature for predicting prognosis in triple‐negative breast cancer
Source: J Cell Mol Med. 2020 Jul 10;24(16):9378–87. doi: 10.1111/jcmm.15588 (PMC7417707; doi:10.1111/jcmm.15588)

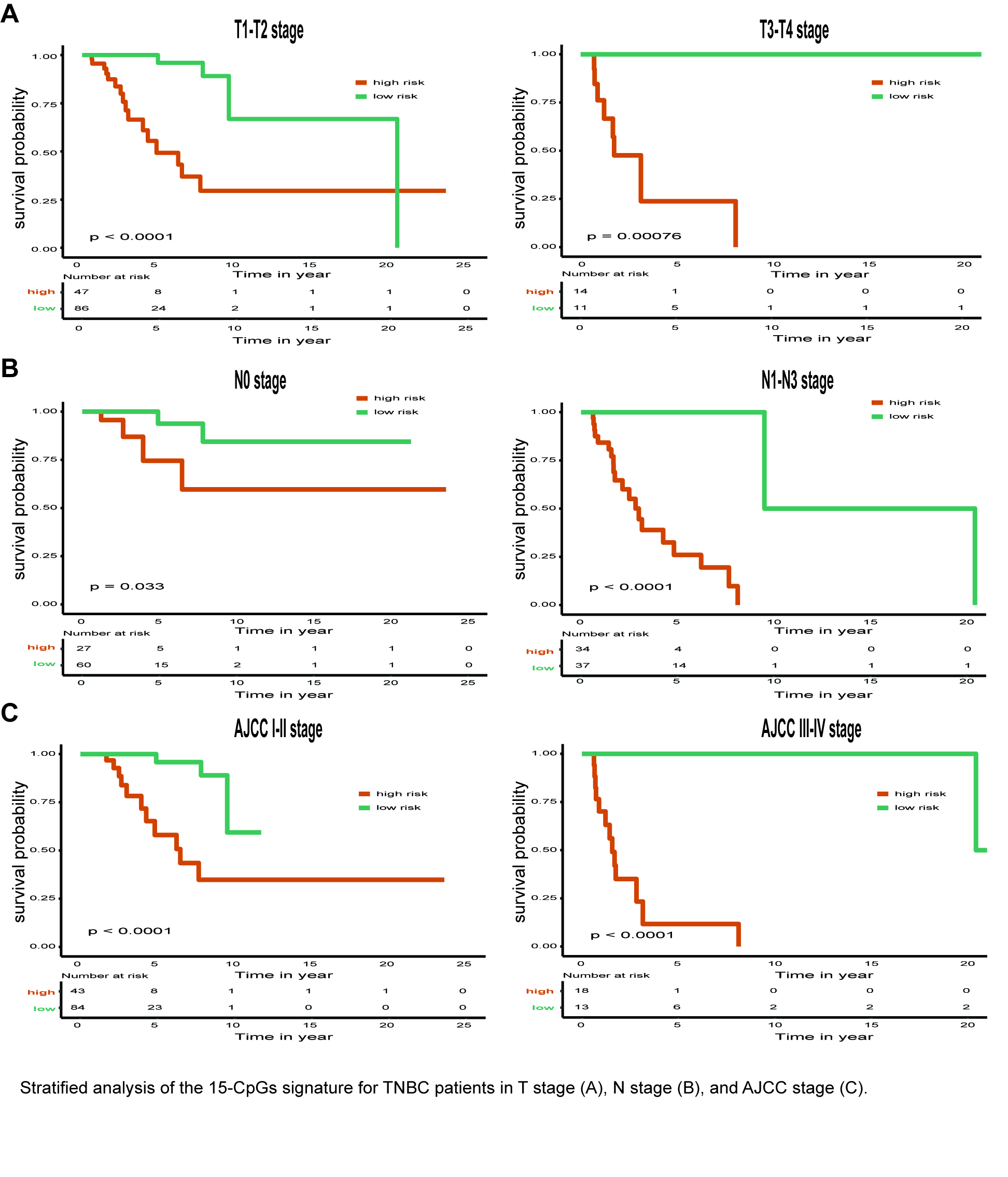

Supplement: Supplementary file 1 — Fig S1 [file JCMM-24-9378-s001.tif]
