# Supplementary material for: Development and validation of a novel 15‐CpG‐based signature for predicting prognosis in triple‐negative breast cancer
Source: J Cell Mol Med. 2020 Jul 10;24(16):9378–87. doi: 10.1111/jcmm.15588 (PMC7417707; doi:10.1111/jcmm.15588)
Supplement: Supplementary file 2 — Tab S1 [file JCMM-24-9378-s002.docx]

**Table S1. Baseline characteristics of study patients.**

|  | **Train set (n=120)** | **Test set (n=38)** | **P-value** |
| --- | --- | --- | --- |
| **Gender** |  |  | 1 |
| Female | 120 (100%) | 38 (100%) |  |
| **Age** |  |  | 0.268 |
| Mean (SD) | 54.7 (12.3) | 57.2 (12.5) |  |
| **Race** |  |  | 0.261 |
| Non white | 49 (40.8%) | 12 (31.6%) |  |
| White | 67 (55.8%) | 26 (68.4%) |  |
| NA | 4 (3.3%) | 0 (0%) |  |
| **Surgical procedure** |  |  | 0.43 |
| Modified Radical Mastectomy | 32 (26.7%) | 13 (34.2%) |  |
| Other | 79 (65.8%) | 24 (63.2%) |  |
| NA | 9 (7.5%) | 1 (2.6%) |  |
| **Histological type** |  |  | 0.208 |
| Infiltrating Ductal Carcinoma | 94 (78.3%) | 31 (81.6%) |  |
| Infiltrating Lobular Carcinoma | 4 (3.3%) | 2 (5.3%) |  |
| Other | 22 (18.3%) | 4 (10.5%) |  |
| NA | 0 (0%) | 1 (2.6%) |  |
| **Menopause status** |  |  | 0.764 |
| Post | 86 (71.7%) | 25 (65.8%) |  |
| Pre | 25 (20.8%) | 10 (26.3%) |  |
| NA | 9 (7.5%) | 3 (7.9%) |  |
| **Margin status** |  |  | 0.464 |
| Negative | 102 (85.0%) | 32 (84.2%) |  |
| Positive | 10 (8.3%) | 5 (13.2%) |  |
| NA | 8 (6.7%) | 1 (2.6%) |  |
| **T stage** |  |  | 0.907 |
| T1 | 28 (23.3%) | 9 (23.7%) |  |
| T2 | 71 (59.2%) | 25 (65.8%) |  |
| T3 | 16 (13.3%) | 3 (7.9%) |  |
| T4 | 3 (2.5%) | 1 (2.6%) |  |
| TX | 1 (0.8%) | 0 (0%) |  |
| NA | 1 (0.8%) | 0 (0%) |  |
| **N stage** |  |  | 0.334 |
| N0 | 64 (53.3%) | 22 (57.9%) |  |
| N1 | 43 (35.8%) | 9 (23.7%) |  |
| N2 | 8 (6.7%) | 4 (10.5%) |  |
| N3 | 4 (3.3%) | 2 (5.3%) |  |
| NX | 0 (0%) | 1 (2.6%) |  |
| NA | 1 (0.8%) | 0 (0%) |  |
| **M stage** |  |  | 0.602 |
| M0 | 99 (82.5%) | 31 (81.6%) |  |
| M1 | 2 (1.7%) | 2 (5.3%) |  |
| MX | 18 (15.0%) | 5 (13.2%) |  |
| NA | 1 (0.8%) | 0 (0%) |  |
| **AJCC stage** |  |  | 0.986 |
| I | 18 (15.0%) | 5 (13.2%) |  |
| II | 79 (65.8%) | 25 (65.8%) |  |
| III | 19 (15.8%) | 6 (15.8%) |  |
| IV | 2 (1.7%) | 1 (2.6%) |  |
| NA | 2 (1.7%) | 1 (2.6%) |  |
| **Survival time** |  |  |  |
| Mean (SD) | 3.57 (3.49) | 4.31 (4.22) | 0.335 |
| Median [Min, Max] | 2.43 [0.0932, 23.6] | 2.69 [0.367, 20.4] |  |
| **Survival state** |  |  |  |
| alive | 99 (82.5%) | 31 (81.6%) | 1 |
| dead | 21 (17.5%) | 7 (18.4%) |  |

*Abbreviations: AJCC American Joint Committee on Cancer*
