# Supplementary material for: Development and validation of a novel 15‐CpG‐based signature for predicting prognosis in triple‐negative breast cancer
Source: J Cell Mol Med. 2020 Jul 10;24(16):9378–87. doi: 10.1111/jcmm.15588 (PMC7417707; doi:10.1111/jcmm.15588)
Supplement: Supplementary file 3 — Tab S2 [file JCMM-24-9378-s003.docx]

**Table S2. Baseline characteristics of study patients between normal and TNBC.**

|  | **normal (N=98)** | **TNBC (N=158)** | **P-value** |
| --- | --- | --- | --- |
| **gender** |  |  | 1 |
| Female | 98 (100%) | 158 (100%) |  |
| **race** |  |  | <0.001 |
| Non_white | 5 (5.2%) | 61 (39.0%) |  |
| White | 90 (93.8%) | 93 (59.1%) |  |
| NA | 3 (1.0%) | 4 (1.9%) |  |
| **age** |  |  | 0.201 |
| Mean (SD) | 57.6 (15.3) | 55.3 (12.5) |  |
| Median [Min, Max] | 57.0 [28.0, 90.0] | 53.5 [28.0, 90.0] |  |
| **Surgical procedure** |  |  | 1 |
| Modified Radical Mastectomy | 27 (28.1%) | 43 (27.9%) |  |
| Other | 56 (58.3%) | 102 (66.2%) |  |
| NA | 15 (13.5%) | 11 (5.8%) |  |
| **Histological type** |  |  | 0.551 |
| Infiltrating Ductal Carcinoma | 78 (81.0%) | 121 (78.6%) |  |
| Infiltrating Lobular Carcinoma | 6 (6.1%) | 6 (3.9%) |  |
| Other | 12 (12.4%) | 26 (16.9%) |  |
| NA | 2 (0.5%) | 3 (0.6%) |  |
| **menopause_status** |  |  | 0.13 |
| Indeterminate | 1 (1.0%) | 3 (1.9%) |  |
| Peri | 2 (2.1%) | 8 (5.2%) |  |
| Post | 54 (56.2%) | 102 (66.2%) |  |
| Pre | 25 (26.0%) | 30 (19.5%) |  |
| NA | 16 (14.6%) | 13 (7.1%) |  |
| **margin_status** |  |  | 0.00124 |
| Close | 2 (2.1%) | 9 (5.8%) |  |
| Negative | 67 (69.8%) | 132 (85.7%) |  |
| Positive | 2 (2.1%) | 6 (3.9%) |  |
| Missing | 27 (26.1%) | 9 (4.5%) |  |
| **T stage** |  |  | 1 |
| T1 | 22 (22.9%) | 37 (24.0%) |  |
| T2 | 61 (63.5%) | 95 (61.7%) |  |
| T3 | 9 (9.4%) | 19 (12.3%) |  |
| T4 | 4 (4.2%) | 3 (1.9%) |  |
| TX | 2 (0.02%) | 2 (0.01%) |  |
| **N stage** |  |  | 0.0824 |
| N0 | 37 (38.5%) | 85 (55.2%) |  |
| N1 | 44 (45.8%) | 51 (33.1%) |  |
| N2 | 8 (8.3%) | 11 (7.1%) |  |
| N3 | 4 (4.2%) | 6 (3.9%) |  |
| NX | 5 (3.1%) | 3 (0.6%) |  |
| **M stage** |  |  |  |
| M0 | 90 (93.8%) | 129 (83.8%) | 0.0643 |
| M1 | 1 (1.0%) | 3 (1.9%) |  |
| MX | 7 (5.2%) | 24 (14.3%) |  |
| **Ajcc pathologic tumor stage** |  |  | 0.711 |
| I | 14 (14.6%) | 23 (14.9%) |  |
| II | 61 (63.5%) | 104 (67.5%) |  |
| III | 20 (20.8%) | 24 (15.6%) |  |
| IV | 1 (1.0%) | 3 (1.9%) |  |
| NA | 2 (0.02%) | 2 (0.01%) |  |
| **Survival state** |  |  | 0.00145 |
| alive | 63 (64.6%) | 129 (83.1%) |  |
| dead | 35 (35.4%) | 28 (16.9%) |  |
| **Survival time** |  |  | 0.283 |
| Mean (SD) | 1420 (873) | 1280 (1150) |  |
| Median [Min, Max] | 1190 [34.0, 3960] | 910 [34.0, 8610] |  |
