# Supplementary material for: Development and validation of a novel 15‐CpG‐based signature for predicting prognosis in triple‐negative breast cancer
Source: J Cell Mol Med. 2020 Jul 10;24(16):9378–87. doi: 10.1111/jcmm.15588 (PMC7417707; doi:10.1111/jcmm.15588)
Supplement: Supplementary file 4 — Tab S3 [file JCMM-24-9378-s004.docx]

**Table S3. 15 CpGs significantly associated with OS in the primary cohort**

| **CpG point** | **Chromosome** | **Start** | **End** | **Symbol** | **HR** | **95% CI** | **P value** | **Lasso Coefficient** |
| --- | --- | --- | --- | --- | --- | --- | --- | --- |
| **ch.16.13300564R** | chr16 | 13299206 | 13299206 | U91319.1 | 170000 | (250-1.2e+08) | <0.001 | 1.90527225 |
| **cg06097659** | chr1 | 1206611 | 1206612 | TNFRSF18 | 23000 | (120-4600000) | <0.001 | 0.703521905 |
| **cg06088745** | chr16 | 54283501 | 54283502 | IRX3 | 17 | (3.3-85) | <0.001 | 1.852205037 |
| **cg24134219** | chr5 | 76082351 | 76082352 | CTC-235G5.3 | 17 | (1.4-190) | 0.026 | 1.101909447 |
| **cg00640314** | chr8 | 66923618 | 66923619 | SNHG6 | 13 | (2-89) | <0.001 | 0.947731163 |
| **cg16302790** | chr13 | 27924197 | 27924198 | PDX1 | 7.4 | (1.4-39) | 0.019 | 1.578144374 |
| **cg14353137** | chr8 | 66961564 | 66961565 | TCF24 | 7.2 | (1.1-46) | 0.037 | 0.052867579 |
| **cg20305005** | chr2 | 1.65E+08 | 1.65E+08 | SCN2A | 0.076 | (0.0074-0.77) | 0.029 | -0.319385907 |
| **cg26302986** | chr7 | 90662046 | 90662047 | CDK14 | 0.07 | (0.0077-0.63) | 0.017 | -0.0806239 |
| **cg03465280** | chr8 | 1.01E+08 | 1.01E+08 | NACAP1 | 0.064 | (0.0054-0.77) | 0.03 | -0.087948794 |
| **cg04598224** | chr19 | 11673699 | 11673700 | ZNF833P | 0.06 | (0.0058-0.61) | 0.017 | -1.238478759 |
| **cg03162410** | chr19 | 14560610 | 14560611 | TECR | 0.0019 | (7.4e-05-0.049) | <0.001 | -4.128076721 |
| **cg01785719** | chr8 | 1.42E+08 | 1.42E+08 | TSNARE1 | 0.00039 | (4.8e-06-0.033) | <0.001 | -0.597341587 |
| **cg14531093** | chr14 | 1.03E+08 | 1.03E+08 | ANKRD9 | 6.20E-06 | (4.7e-10-0.082) | 0.013 | -5.56676195 |
| **cg23023126** | chr19 | 308808 | 308809 | MIER2 | 4.10E-11 | (1.8e-16-9.6e-06) | <0.001 | -3.784874857 |

*Abbreviations: OS, overall survival; CI, confidence interval; Lasso Lasso Least Absolute Shrinkage and Selector Operation.*
