# Supplementary material for: Development and validation of a novel 15‐CpG‐based signature for predicting prognosis in triple‐negative breast cancer
Source: J Cell Mol Med. 2020 Jul 10;24(16):9378–87. doi: 10.1111/jcmm.15588 (PMC7417707; doi:10.1111/jcmm.15588)
Supplement: Supplementary file 5 — Tab S4 [file JCMM-24-9378-s005.docx]

**Tabel S4. Univariate and multivariate analyses of clinicopathological characteristics**

|  | **Univariate analysis** |  | **Multivariate analysis** |  |
| --- | --- | --- | --- | --- |
| **Variables** | **HR (95% CI)** | **P value** | **HR (95% CI)** | **P value** |
| Age | 0.809(0.384-1.706) | 0.577 |  |  |
| race | 0.539(0.25-1.161) | 0.114 |  |  |
| Surgical procedure | 0.645(0.297-1.401) | 0.268 |  |  |
| Menopause status | 0.611(0.254-1.472) | 0.273 |  |  |
| Margin status | 2.195(0.884-5.448) | 0.09 |  |  |
| T stage | 2.208(0.967-5.042) | 0.06 |  |  |
| N stage | 4.843(1.957-11.983) | < 0.001 |  |  |
| M stage | 4.759(1.524-14.859) | 0.007 |  |  |
| AJCC stage | 4.478(2.111-9.496) | < 0.001 | 5.271(2.463-11.28) | < 0.001 |
| Risk score | 13.82(4.777-40.01) | < 0.001 | 16.370(5.511-48.63) | < 0.001 |

AJCC American Joint Committee on Cancer; HR, Hazard ratios; CI, confidence interval
