# Supplementary material for: Development and validation of a novel 15‐CpG‐based signature for predicting prognosis in triple‐negative breast cancer
Source: J Cell Mol Med. 2020 Jul 10;24(16):9378–87. doi: 10.1111/jcmm.15588 (PMC7417707; doi:10.1111/jcmm.15588)
Supplement: Supplementary file 6 — Tab S5 [file JCMM-24-9378-s006.docx]

|  | **Univariable or Multivariable** | | **C-index** (95%CI) |
| --- | --- | --- | --- |
| **Model1*** | HR (95%CI) | P Value | 0.918 (0.896-0.940) |
| **Risk score** |  |  |  |
| Low | 1 [Reference] |  |  |
| High | 30.72 (8.32-113.4) | <0.001 |  |
| **AJCC stage** |  |  |  |
| I | 1 [Reference] |  |  |
| II | 16.23 (1.72-153.0) | 0.015 |  |
| III | 40.67 (4.41-375.0) | 0.001 |  |
| IV | 92.57 (8.86-966.9) | <0.001 |  |
| **Model2*** |  |  | 0.789 (0.746-0.832) |
| **AJCC stage** |  |  |  |
| I | 1 [Reference] |  |  |
| II | 3.89 (0.51-29.79) | 0.191 |  |
| III | 12.74 (1.63-99.68) | 0.015 |  |
| IV | 22.66 (2.47-208.06) | 0.006 |  |
| **Model3*** |  |  |  |
| **Risk score** |  |  | 0.819 (0.799-0.839) |
| Low | 1 [Reference] |  |  |
| High | 13.82 (4.77-40.01) | <0.001 |  |

**Table S5. Comparison of different clinical models**

*Model1*: Based on AJCC stage and risk score; Model2*: Based on AJCC stage; Model3*: Based on risk score.*
